# OLDER ADULT ONLINE EXERCISE CLASSES DURING THE COVID-19 PANDEMIC: A SURVEY OF SERVICE PROVIDER PERSPECTIVESS

**DOI:** 10.1093/geroni/igac059.2885

**Published:** 2022-12-20

**Authors:** Dallas Murphy, Michelle Porter, Ruth Barclay, Stephen Cornish, Jacquie Ripat, Kathryn Sibley, Sandra Webber, Nicole Dunn

**Affiliations:** University of Manitoba, WINNIPEG, Manitoba, Canada; University of Manitoba, Winnipeg, Manitoba, Canada; University of Manitoba, Winnipeg, Manitoba, Canada; University of Manitoba, Winnipeg, Manitoba, Canada; University of Manitoba, Winnipeg, Manitoba, Canada; University of Manitoba, Winnipeg, Manitoba, Canada; University of Manitoba, Winnipeg, Manitoba, Canada; University of Manitoba, Winnipeg, Manitoba, Canada

## Abstract

COVID-19 rendered the availability of exercise facilities sporadic and online exercise programs subsequently became more common. This research explored online exercise classes delivered to older adults during the pandemic from the perspective of service providers. Sixty-seven service providers completed the survey (88% female). The majority (54%) of respondents had worked in the fitness industry for greater than 10 years, and 66% were fitness class instructors, while fewer were managers (9%) and personal trainers (8%). Three participants had experience providing online exercise classes prior to the pandemic, while 43 more had experience providing online exercise classes since the pandemic began. Of these 46 service providers, 87% offered classes live through Zoom. The majority (64%) offered classes through an organization, and 61% charged a fee for participants to take part. The most common type of class was a general fitness class (63%), followed by yoga and flexibility classes (39%), and strength training (17%). Regarding equipment used, weights were most frequently required (69%), followed by resistance bands (49%) and mats (44%). Most classes lasted 40–60 minutes (59%) and were low intensity (74%). Of the 21 respondents who did not provide online exercise classes, 43% indicated this was because of a lack of interest, and 19% cited not knowing how to use technology to deliver classes online, though most (71%) indicated they would consider offering online classes in the future. This research reveals the adaptability of service providers and may serve to inform the continued development of online exercise programs for older adults.

